# Functional and structural studies of tolloid-like 1 mutants associated with atrial-septal defect 6

**DOI:** 10.1042/BSR20180270

**Published:** 2019-01-08

**Authors:** Lukasz Sieron, Marta Lesiak, Izabela Schisler, Zofia Drzazga, Andrzej Fertala, Aleksander L. Sieron

**Affiliations:** 1 Department of Molecular Biology and Genetics, School of Medicine in Katowice, Medical University of Silesia in Katowice, Katowice, Poland; 2 Department of Medical Physics, Institute of Physics, University of Silesia, The Silesian Centre for Education and Interdisciplinary Research, Chorzów, Poland; 3 Department of Orthopedic Surgery, Sidney Kimmel Medical College, Thomas Jefferson University, Philadelphia, PA, U.S.A.

**Keywords:** atrial-septal defect, endopeptidase activity, mammalian tolloid-like 1, point mutation, protein structure, tolloid

## Abstract

Inactive *mammalian tolloid-like 1* (*tll1*) and mutations detected in tolloid-like 1 (*TLL1*) have been linked to the lack of the heart septa formation in mice and to a similar human inborn condition called atrial-septal defect 6 (ASD6; OMIM 613087, formerly ASD II). Previously, we reported four point mutations in *TLL1* found in approximately 20% of ASD6 patients. Three mutations in the coding sequence were M182L, V238A, and I629V. In this work, we present the effects of these mutations on TLL1 function. Three recombinant cDNA constructs carrying the mutations and one wild-type construct were prepared and then expressed in HT-1080 cells. Corresponding recombinant proteins were analyzed for their metalloendopeptidase activity using a native substrate, chordin. The results of these assays demonstrated that in comparison with the native TLL1, mutants cleaved chordin and procollagen I at significantly lower rates. CD analyses revealed significant structural differences between the higher order structure of wild-type and mutant variants. Moreover, biosensor-based assays of binding interactions between TLL1 variants and chordin demonstrated a significant decrease in the binding affinities of the mutated variants. The results from this work indicate that mutations detected in *TLL1* of ASD6 patients altered its metalloendopeptidase activity, structure, and substrate-binding properties, thereby suggesting a possible pathomechanism of ASD6.

## Introduction

Mammalian tolloid-like 1 (TLL1) is classified in the evolutionarily conserved family of zinc-dependent metalloendopeptidases. These metalloendopeptidases are also known as tolloids (BMP1/TLD/Tolloid-like). They are present across the animal world, including humans [1]. The first discovered tolloid of the family in mammals was BMP1 [2], also known as procollagen C-endopeptidase, which engages in the formation of the extracellular matrix (ECM) [3,4]. BMP1 and the longer alternatively spliced variant of the *BMP1* gene product, TLD [5], demonstrate high homology to a protein named *Drosophila* tolloid-related (tlrd) in fruit flies. The *Drosophila* tlrd is involved in embryonal signaling and development, leading to the designation of the dorsal–ventral body axis [6]. Another protein, tolloid-related protein 1 (TLR1), is a key factor in transformation of the fly larvae [7,8]. These homologous proteins are involved in both the formation of the ECM and in numerous signaling pathways [9–16]. Four tolloid proteins have been detected in mammals thus far [16,17]. Two of them, BMP1 and TLD1, are products of the alternative splicing of the *BMP1* gene product. The two other proteins (mammalian tolloid-like 1 [TLL1] and mammalian tolloid-like 2 [TLL2]) are encoded by separate genes [16–18].

Studies in transgenic mice with the knockout of *tll1* gene (*tll1*^−/−^) revealed that the presence of this metalloendopeptidase is critical for the formation of septa in the murine heart. The homozygous silencing of this gene was lethal in the mice, leading to their death at mid-gestation due to defects in the blood circulatory system related to the lack of its proper flow because of the ventricular septal defect (VSD) and atrial-septal defect (ASD), which were accompanied by dysplasia of the mitral valve [19,20]. Less common defects, such as a double outlet of the right ventricle and a two-upstream chamber left ventricle, were also observed [19].

In our previous work, we linked mutations in *TLL1* to human atrial-septal defect 6 (ASD6) (OMIM 613087) [21]. Although the exact molecular mechanism of the regulation of heart development by TLL1 is not clear, studies suggest its ability to cleave chordin play a substantial role [18,19].

The leading signaling path in which TLL1 may be involved in heart development seems to include BMP2 and BMP4 [22,23]. TLL1 cleaves chordin, which binds to BMP2 and BMP4, then releases these morphogens and allows them to interact with their cell surface receptors. BMP2 is an important factor in the process of the epithelial–mesenchymal transition (EMT) within the endocardial panels [24]. An artificial distribution of BMP2 within the axial mesoderm induced its differentiation into cardiac lines of the constituent cells. In contrast, an introduction of Noggin, an inhibitor of BMP2 and BMP4, into the heart of chick embryos reduced the expression of genes that are markers of cardiogenesis such as *NKX2.5, GATA4, eHAND*, and *Mef2A* [18]. The interaction of BMP2, SMAD1, and NKX2.5 forms a self-regulating feedback loop that affects the differentiation and proliferation of the cells within the secondary heart-forming field [25]. BMP4 induces differentiation of progenitor cells into cardiomyocytes by reducing the expression of *ISL1* and *TBX1* genes [26]. Inhibition of BMP4 expression leads to a reduction in the number of the endocardial cushion-forming cells, which in the later stage of development is manifested by partial interventricular septum formation, common arterial trunk, and the semilunar valve defect [27]. That is why the regulation of BMP2/4 signaling seems to be the most suited to the role of the *tll-1* gene in the creation and development of the septa in the murine heart [27].

Therefore, the question is whether the mechanisms discovered in the murine model are relevant to human ASD6. *TLL1* in humans is located in the chromosomal region 4q32–q33 [28]. Deletions within this region are associated with congenital heart defects such as ASD and VSD [29]. Therefore, in our previous work, we screened the *TLL1* sequence for mutations in patients with ASD6, formerly called ASDII [21]. Three individual missense mutations were detected. Two of those mutations were homozygous mutations changing the amino acids Met182Leu and Val238Ala. The third mutation was a heterozygous change of Ile629Val. These mutations were located at sites that may affect TLL1 enzymatic activity. Amino acids 182 and 238 are located in the catalytic domain, and thus they might have a direct impact on enzyme cleavage activity. In contrast, the third change located in the C-terminal CUB3 domain is postulated to change the affinity of the enzyme to the substrate [21]. However, at the time of the mutations’ detection, their effects on enzyme activity were not verified. Therefore, four recombinant proteins, the three mutants and the native one, were prepared and tested for changes in their structures and functional effects by means of enzymatic activity: on chordin, to determine cleavage rates; on procollagen type I, to determine substrate specificity of the mutants; and on a synthetic peptide, to detect the relation between the mutations and their proteolytic action.

## Materials and methods

### Cell cultures

For the production of recombinant proteins, human fibrosarcoma HT-1080 (ATCC, CCL-121TM) cells were employed, as described [4]. The cell line expressing human *TLL1* was derived from NTERA-2 cells, which were derived from a human embryonic teratoma (ATCC, HTB-106). Conditions for their culture were the same as for the HT-1080 cells.

### Preparation of the genetic constructs for *in vitro* expression

DNA constructs for expression of recombinant proteins in HT-1080 cells were cloned into the pIRESneo3 vector (Clontech, U.S.A.) containing the CMV promoter and the polyadenylation site sequences. The cassette vector also contained an Internal Ribosome Entry Site sequence (IRES) followed by the gene sequence coding for neomycin resistance.

A fragment of cDNA encoding functional TLL1 enzyme, lacking the signal peptide and the latency pro-domain (nucleotides 1089–3686 of the reference sequence accession number NM_012464.4 GenBank database mRNA variant 1 *TLL1*) was combined with a sequence encoding a signal peptide of the α 1 chain of type I procollagen (nucleotides 5127–5192, reference sequence accession number NM_007400.1 GenBank database mRNA *COL1A1*, LRG 1). This combination of sequences has previously proven efficient for secretion of recombinant BMP1/TLD1 from HT-1080 cells into the culture medium [30]. To facilitate the purification of recombinant proteins from the culture medium, a sequence encoding a 7xhistidine-tag at the C-terminus of the TLL1 was included.

For the preparation of the cDNA encoding human TLL1 the total RNA from the NTERA-2 cells cultured in the 175 cm^2^ flask was isolated by a standard TRIzol/chloroform method (Sigma–Aldrich, Germany) and subjected to a standard RNA purification procedure. Subsequently, 5 μg of total RNA was subjected to a reverse transcription reaction using the d’ART set according to the manufacturer’s protocol (EURx, Poland). To increase the amount of TLL1 cDNA, a PCR with the use of primers (shown in Supplementary Table S1) (Oligo.pl, Poland) designed with the help of PrimerBlast software (www.ncbi.nlm.nih.gov) was performed.

The PCR was conducted by using reagent with Fast Start polymerase (Roche) according to the manufacturer’s instructions. The reaction mixture was incubated in the thermocycler Applied Biosystems GeneAmpPCR system 9700 (Life Technologies, U.S.A.) with a temperature profile recommended by the polymerase supplier.

The PCR product was detected following electrophoresis in 1.5% agarose gel (Sigma–Aldrich, Germany) in TAE buffer (Labempire, Poland). Due to the expected low level of *TLL1* expression and the long stretch of amplified DNA, the cDNA fragment containing the sequence coding for protein was divided into fragments: A, comprising nucleotides 1090–2790 and B, comprising nucleotides 2770–3686 both in the reference sequence accession number NM_012464.4 GenBank database mRNA variant 1 *TLL1*. For amplification, a two-stage nested PCR was performed. In the first PCR, the primers used were TA1F and TSR7 (Supplementary Table S1) for the A fragment and TSF3 and TA1BR primers (Supplementary Table S1) for the B fragment. The second PCR-amplifying target segment was conducted using 2 μl of template from the previous PCR and the primer pair FASTCOL and RW1SECO (Supplementary Table S1) for the A fragment and FW1SECO and RW1HISN primers (Supplementary Table S1) for the B fragment.

The fragment C comprising the sequence encoding the procollagen I α 1 chain signal peptide (nucleotides 5127–5192, sequence accession number NM_007400.1 GenBank database mRNA *COL1A1*, LRG 1) was amplified with COLZ and RCOLAST primers (Supplementary Table S1) by PCR with 100 ng of genomic DNA template purified from human fibroblasts with the wild-type *COL1A1* gene sequence.

The PCR products were purified using a PCR/DNA clean-up kit (EURx, Poland). Purified fragment B was cleaved with *Eco*RI and *Not*I (EURx, Poland) according to the manufacturer’s protocol, re-purified, and cloned into the pIRESneo3 vector (Clontech, U.S.A.). The obtained plasmid pIRESneo3-B was propagated in TOP10 *Escherichia coli* strain (Invitrogen, U.S.A.). Isolation of plasmid DNA was carried out using a Plasmid Miniprep DNA Purification Kit (EURx, Poland). Fragments A and C were cleaved with *N*arI (EURx), purified, and ligated together. The product of ligation was amplified by PCR with primers FCW1PIN and RW1SECO (Supplementary Table S1). Amplified fragment C–A was purified, cleaved with *Pin*AI and *Eco*RI (EURx), and cloned into the pIRESneo3-B vector.

The sequencing was performed by a semi-automatic Sanger’s method to verify the correct DNA sequence of the prepared fragments. For sequencing TSF1-8 and TSR1-8, as well as for IRSEQF and NEO3R, primers complementary to inserting flanking plasmid sequences were used. The sequencing reaction products were analyzed using the capillary DNA Analyzer ABI Prism 3130xl Applied Biosystems (U.S.A.). The sequences obtained were compared with the reference sequence in the APE-A plasmid Editor v2.0.45.

### Mutagenesis of the cDNAs

Mutagenesis was conducted using the construct for amplification with primers introducing the mutation, and subsequent degradation of the template by its digestion with restriction endonuclease *Dpn*I (EURx). For mutagenesis, the primer pairs M1R and M1F (Supplementary Table S1) introduced mutation 1, M2F and M2R (Supplementary Table S1), introduced mutation 2 and M3F and M3R (Supplementary Table S1), and introduced mutation 3. For the PCR, the polymerase KapaHiFi premix (Cope Biosystems, U.S.A.) was used.

The degradation of the matrix of the plasmid was performed by incubation of 10 μl of PCR mixture with *Dpn*I (EURx) according to the manufacturer’s protocol. To transform the bacteria, 5 μl of reaction mixture was used. The resulting colonies were analyzed for the presence of introduced mutations following expansion of five colonies and subsequent plasmid isolation and DNA sequencing. Constructs with the introduced mutation but without other sequence changes were used for transfection of HT-1080 cells.

### Transfection of HT-1080 cells

One day prior to transfection, approximately 3 × 10^5^ of HT-1080 cells in a volume of 2 ml of complete DMEM medium were seeded into each well of a six-well plate. The next day, 12 μl of reagent ‘K2 multiplier’ (Biontex, Germany) was added to the cell medium and incubated for 2 h under standard culture conditions for HT-1080 cells. Then, the plasmid DNA transfection was carried out with 2 μg of plasmid DNA complexed with 5 μl of K2 reagent in 200 μl DMEM, as recommended by the supplier. Complexes were added to the medium, and the cells were seeded with the complexes evenly distributed across the culture plate. The cells were incubated with the DNA overnight under standard culture conditions. Subsequently, the cells were detached following trypsin digestion and plated in 10-cm diameter cell culture dishes. The following day, selection for stably transfected cells was started by replacing the medium with medium supplemented with 300 μg/ml of G418. The selection medium was changed every other day. The antibiotic-resistant clones were detached and passaged for further expansion and testing for recombinant protein production.

### Detection of recombinant proteins production and its secretion into the cell culture medium

The culture medium (1.0 ml) collected from each of the tested clones, at 70% confluence, was centrifuged in order to remove cell debris. Subsequently, the sample was concentrated using Amicon filters fitted with a PVDF membrane (30 kDa) according to the manufacturer’s protocol (Amicon). The condensed samples as well as samples from lysed cells in order to ensure that mutations did not affect TLL1 secretion were analyzed by Western blot following SDS/PAGE of the concentrated proteins separated on mini gels using a Protean electrophoresis system (Bio-Rad, U.S.A.) with a 4% stacking and an 8% separating SDS/polyacrylamide gel. Samples were mixed with standard loading buffer (Pierce) supplemented with 1% β-mercaptoethanol and heat denatured for 5 min at 95°C. The molecular weight marker was a 4-μl mixture of proteins with known size (Kaleidoscope; Bio-Rad, U.S.A.). After the electrophoresis was completed, the proteins were electrophoretically transferred on to PVDF membrane (Immobilon-P Transfer Membrane, Millipore Merck, Germany). Subsequently, the membranes were incubated with rat monoclonal antibodies against the C-terminal sequence of the human TLL1 (Biorbyt, England) at a dilution of 1:2000 and then to 1:15000 diluted secondary anti-rabbit IgG antibodies (IgG Whole Molecule) conjugated to horseradish peroxidase (Horseradish Peroxidase Conjugated Sigma–Aldrich). The secondary antibodies were identified as a result of a chemiluminescent reaction with the catalyst (Quantum Company Advansta, U.S.A.). The membrane was exposed to light-sensitive film (Hyperfilm, GE Healthcare) for 10 min and developed.

### Production and purification of recombinant TLL1 and its mutated variants

Stably transfected HT-1080 clones were expanded into twelve 175-cm^2^ tissue culture flasks.

The collection of serum-free culture medium containing the recombinant protein began when cells reached 70% culture confluence. The cell layer was rinsed three times with 15 ml of pre-warmed PBS and 15 ml of fresh serum-free DMEM medium (Sigma–Aldrich) supplemented with 1% antibiotic/antimicotic (PAA Laboratories GmbH, Austria), and 2 mM l-glutamine (PAA Laboratories GmbH, Austria) was added. The cells were incubated for two consecutive days, regularly inspecting the culture status. The collected medium was centrifuged to separate floating debris and dead cells, then frozen at −70°C, and stored until further use.

After three consecutive collections, complete DMEM was added for 24 h in order to provide better nutrition and ‘regenerate’ the cells. The harvest procedure was repeated. The medium with the recombinant protein was harvested at least six times from each culture.

Recombinant proteins produced and secreted by HT-1080 cells into the culture medium were purified utilizing the affinity of the 7xhistidine tag at the carboxyl terminus of the recombinant protein to the nickel resin. Therefore, to approximately 500 ml of the harvested culture medium, Trizma-base and NaCl were added at final concentrations of 50 mM and 0.5 M, respectively. The pH was then adjusted to 8.0 with HCl (Sigma–Aldrich). For Ni^+^-resin purification, 2 ml of balanced bed HisPur Ni-NTA (Thermo Scientific) was added to the protein solution and incubated on the rotator overnight at 4°C. On the following day, the suspension was poured into a hollow housing chromatographic column (Bio-Rad, U.S.A.) 15 cm long and 1.5 cm in diameter. After passing through the column, the whole medium remaining on the column was rinsed with ten bed volumes of washing buffer (50 mM Tris pH 7.5, 0.5 M NaCl). Then, the proteins bound to the resin were eluted with a buffer containing 250 mM imidazole in three fractions of one column volume.

The protein purity was verified following SDS/PAGE and Coomassie Blue gel staining. The samples, the polyacrylamide gels, and the electrophoresis were prepared as already described and analyzed following staining with Coomassie Brilliant Blue G–Colloidal Concentrate (Sigma–Aldrich). Stained gels were dried on cellophane foil and photographed with a digital camera.

Purified protein was concentrated and dialyzed into Assay Buffer (AB) (50 mM Tris pH 7.5, 150 mM NaCl, 5 mM CaCl_2_) using Amicon Ultra-0.5 filters (Millipore) with a cutoff of 30 k. The proteins in the AB were stored at −70°C.

The protein concentration was assayed after electrophoresis of 5 µl of the samples on an 8% polyacrylamide gel. On the same gel, the concentration curve of 25, 50, 75, 125, and 250 μg/ml in a volume of 5 μl were prepared as quantity controls. The gel was stained with Coomassie Brilliant Blue and prepared as already described. The gel was scanned and bands corresponding to either the recombinant protein or diluted BSA were measured for their density. The amount of protein was calculated by linear regression using the linear function pattern derived from the values for the density of bands corresponding to BSA concentration.

### Assays of TLL1 and its mutants’ enzymatic activities

The substrate used for the enzymatic activity was recombinant murine chordin (R&D Systems, U.S.A.). The substrate was dissolved in 250 μl of AB consisting of 50 mM Tris pH 7.5, 150 mM NaCl, 5 mM CaCl_2_, and 0.05% Brij30 to obtain its final concentration 200 µg/ml. Chordin cleavage was carried out in a reaction mixture containing 250 ng chordin and 20 ng of purified TLL1 (at final concentration of 2 ng/µl) enzyme in a final AB volume of 10 μl. The mixture was incubated for 6, 12, 16, and 24 h at 37°C using a GeneAmp thermocycler. To stop the reaction, 2 μl of 5× sample loading buffer containing β-mercaptoethanol was added and the mixture was denatured for 5 min at 95°C. The reaction products were subjected to electrophoresis using an 8% polyacrylamide gel and stained with Coomassie Brilliant Blue as already described. The stained gel was dried, scanned, and analyzed by densitometry of the chordin cleavage product. Only the band corresponding to non-cleaved chordin, marked as ‘Intact’ in Figure 4B, was assayed. The density of the band corresponding to the intact chordin in the reaction mixture with native enzyme was used to calculate the percentile of unprocessed chordin remaining in the reaction mixtures with mutated enzymes. In the case of TLL1 procollagen-type I cutting and its mutant quantitative variants, the measurement of unprocessed procollagen was difficult. The procollagen cleavage products were separated without reduction in SDS/PAGE, therefore, the band corresponding to the uncut triple-helical procollagen I, migrated very slowly and was of poor resolution in the gel image. Therefore, it was impossible to do the exact density measurement. To determine enzymatic activities of TLL1 and its mutated variants on procollagen type I, the densities of bands corresponding to the final cleavage products, marked in the Figure 4C as pNα1(I) and pNα2(I), were measured. The density of the two bands was added and assumed as 100% in the calculations. Similarly, the sum density of corresponding bands generated by mutated variants of TLL1 were obtained and used to calculate the percentile of their wild-type activity. The density of bands was measured with the use of GelQuant.NET software provided by biochemlabsolutions.com.

To verify if the mutation affects the catalytic domain, a synthetic fluorogenic peptide MCA-YVADP-DNP-K was used as a substrate. Substrate concentration, the buffer, and incubation conditions were as for chordin cleavage. Following 4 h of incubation the fluorescence was measured using Fluorodrop 330 (Thermo Scientific, U.S.A.).

Since procollagen type I was shown to be a potential TLL1 substrate [16], it was used to verify if the mutations of interest affected TLL1 substrate specificity. The cleavage was conducted in 10 μl of reaction mixture containing 500 ng of procollagen I and 12 ng of purified TLL1. Other reaction conditions were as already described for cleavage of chordin, here and elsewhere [18]. The reaction was terminated by adding 2 µl of 5× loading buffer (without β-mercaptoethanol) and immediate heat denaturation. The unreduced conditions for SDS/PAGE were used to ensure visualization only the completely cleaved chains. The cleavage rate was determined based on densitometry measurement of the band corresponding to N-propetides of procollagen I α1 and α2 chains. The content of this cleavage product reflects complete removal of the C-propeptides from the three procollagen α chains, whereas, the intermediate products could be either one or two C-propeptides bound via disulphide bridges to still unprocessed procollagen I. Thus, only pN-α chains could be measured as the cleavage products. In the histograms the results were presented as percentage of the wild-type proteolytic activity.

### CD spectrometry of recombinant proteins

For CD spectroscopy, protein concentrations were estimated spectrophotometrically at λ_280 nm_. The samples were denatured with 5 mM guanidine hydrochloride, and the absorbance was measured using the spectrophotometer Nanodrop 2000 (Thermo Fisher Scientific, U.S.A.). Subsequently, each tested sample was prepared at equal concentrations of 0.08 mg/ml in AB. The samples in a volume of 150 μl were loaded into a quartz cuvette with a 0.5-mm pathway length. Measurements were performed using the CD spectrophotometer Jasco J-815 for λ spectrum ranging from 190 to 300 nm at 37°C. The instrument settings were as follows: high sensitivity (5 mdeg), data pitch 0.1 nm, scanning mode continuous, response 1 s.

Spectrum values were exported as an Excel file. The calculation of the content of secondary structures was performed based on neural network theory [31] using CDNN deconvolution software version 2.1 (http://gerald-boehm.de/download/cdnn), which has applicability as a tool for structural molecular biology. The results are represented as percent contribution of α-helices, β-sheets, and random coil structures in the overall structure of the protein in the analyzed sample.

### Interaction of recombinant TLL1 and its mutants with chordin

A biosensor (Sensiq Pioneer, SensiQ Technologies, Inc.) was employed to analyze binding interactions between chordin and TLL1 variants. For these assays, chordin was first bound to the chip surface (COOH1 SPR Chip, SensiQ Technologies, Inc.); carboxylate groups present on the surface were activated by injection of a 1:1 mixture of 0.1 M N-hydroxysuccinimide and 0.4 M N-ethyl-3-(3-dimethylaminopropyl) carbodiimide (Thermo Scientific, Waltham, MA). Subsequently, 100 μg/ml of chordin in 10 mM acetate buffer at pH 4.5 was allowed to bind to the activated surface until a response plateau was reached. The residual active groups were blocked by an injection of 100 μl of 1 M Tris/HCl (pH 8.5). Subsequently, the chip was equilibrated with 4-(2-hydroxyethyl)-1-piperazineethanesulphonic acid (HEPES)-buffered saline containing 0.005% of Tween 20 (HBS-TE). Excess non-bound material was removed by washing the chip with HBS-TE, followed by three consecutive washes with 10 mM HCl. To analyze the kinetics of chordin-TLL1 variants binding, in which chordin served as an acceptor, a sensor chip was primed with HBS-TE at 25°C for 10 min. Subsequently, the association phase was initiated by injecting a free analyte, i.e. a TLL1 variant, at a rate of 10 μl/min for 11 min. After that time, the dissociation phase was initiated by injecting the analyte-free buffer. After each assay, the surface of the sensor chip was regenerated by washing with 10 mM HCl followed by equilibration with HBS-TE. During regeneration cycles, attention was paid to completely remove surface-bound analyte, and the washing continued until a response equal to a baseline value was reached. For binding assays, free TLL1 variants were added at concentrations ranging from 10 to 400 nM. Data from the biosensor were analyzed by the global fitting method described by Morton and Myszka [32]. For each assay, the association rate constants (*k*_on_) and the dissociation rate constants (*k*_off_) were obtained, and the equilibrium dissociation constants (*K*_d_) values were calculated from a ratio of k_off_/k_on_. Subsequently, the equilibrium association constant (*K*_a_) values (termed here as the ‘affinity’) were derived as the inverse of *K*_a_.

### Statistical analysis

All measurements were performed in triplicates or quadruplicates as indicated in the text. Statistical significances of differences were calculated using *t*test in Office Excel software package.

## Results

### Characterization of the cDNA constructs

**Figure 1:**
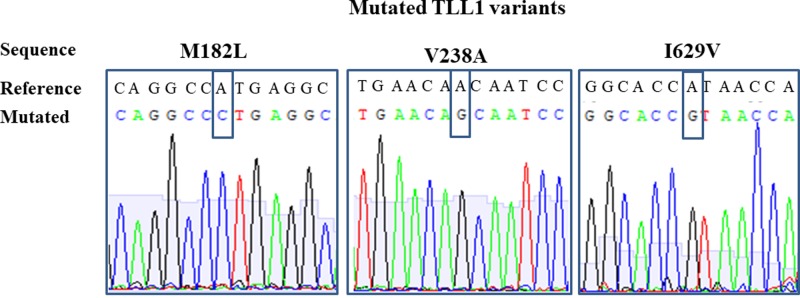
Representative histograms of DNA sequencing of constructs with introduced mutations The mutated nucleotides are framed.

**Figure 2:**
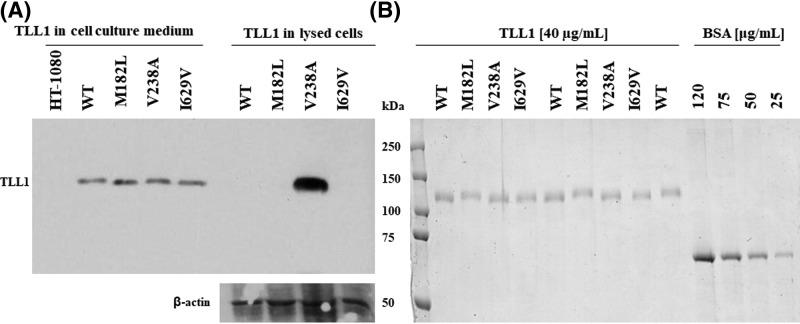
Pattern of WB analysis of proteins secreted into the culture medium and retained inside of selected HT-1080 clones being stable producers of the recombinant TLL1 and its mutated variants (**A**) Detection following Western blot of TLL1 in culture medium of HT-1080 cells that were transfected with plasmids and grown in the presence of G-418. Top picture – detection of TLL1s, bottom picture – detection of β actin. First five lanes from left contained proteins from culture medium of mock transfected HT-1080 (HT-1080), or with plasmid carrying cDNA encoding native TLL1 (WT) and TLL1 with mutations (M182L, V238A, I629V). Next four lanes represent proteins in lysates from the same cells. (**B**) Results of electrophoresis of equal volumes of purified recombinant proteins (40 ng/μl). Lanes contained; WT - native TLL1, M182L, V238A and I629V – mutated variants of TLL1, BSA - bovine serum albumin.

Modified cDNAs encoding TLL1 were cloned into pIRESneo3 to obtain the pIRESneo3-C-TLL-1 plasmid and subjected to mutagenesis that was then verified by sequencing to confirm that the appropriate changes were introduced and to exclude unwanted random changes (Figure 1). The mutated TLL1s’ plasmids were successfully expressed in HT-1080 cells. Their production was detected in all HT-1080 clones producing both the native enzyme or its three mutants except for the mock transfected cells (Figure 2A). TLL1 was also present in HT-1080 cell lysates transfected with mutant V238A in contrast with cells producing native enzyme and the other two mutants, M182L and I629V (Figure 2A). The clones secreting TLL1 variants into the culture medium were used for their production and purification.

### Purification of the recombinant TLL1 and its mutated variants

The proteins were efficiently purified by affinity column chromatography with the use of nickel resin. Based on densitometry measurements of the bands corresponding to purified proteins and bands corresponding to BSA at different known amounts, the concentrations of the purified recombinant enzymes were calculated. The presence of any contaminating proteins were not detected in the samples of recombinant enzymes (Figure 2B). Purified proteins were used for all subsequent tests and measurements based on their structural integrity and substrate-specific activities.

### Analyses of structural properties and activities of the recombinant TLL1 and its mutated variants

**Figure 3:**
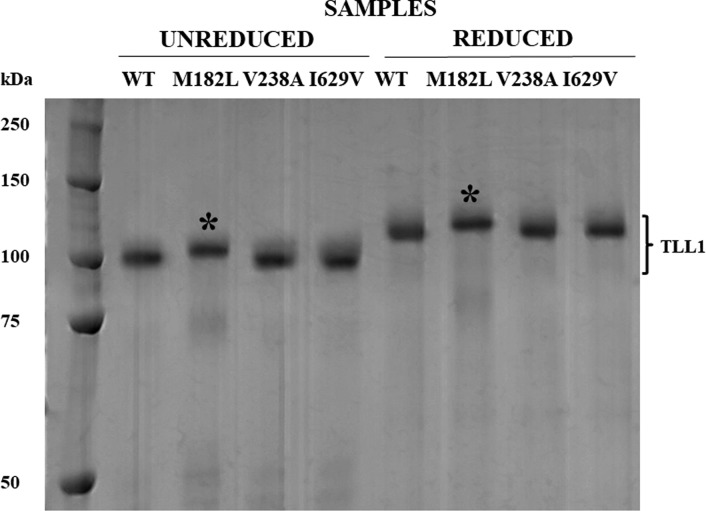
The comparison of migration of purified recombinant TLL1 enzymes following SDS/PAGE Lanes from left: Molecular mass marker, two sets of four lanes each with unreduced samples or reduced samples as in Figure 2B. The asterisks refer to the mutant sample that migrated at a different rate than the other samples.

The samples were subjected to SDS/PAGE separation either non-reduced or reduced with β-mercaptoethanol (Figure 3). Without reduction, the V238A and I629V mutants migrated as the wild-type recombinant protein. However, migration of the mutant M182L was slower. The reduced proteins maintained the pattern of migration except that the migration rate of the M182L mutant was slower (Figure 3).

### Assay of enzymatic activity of TLL1 and its mutated variants on fluorogenic peptide

**Figure 4:**
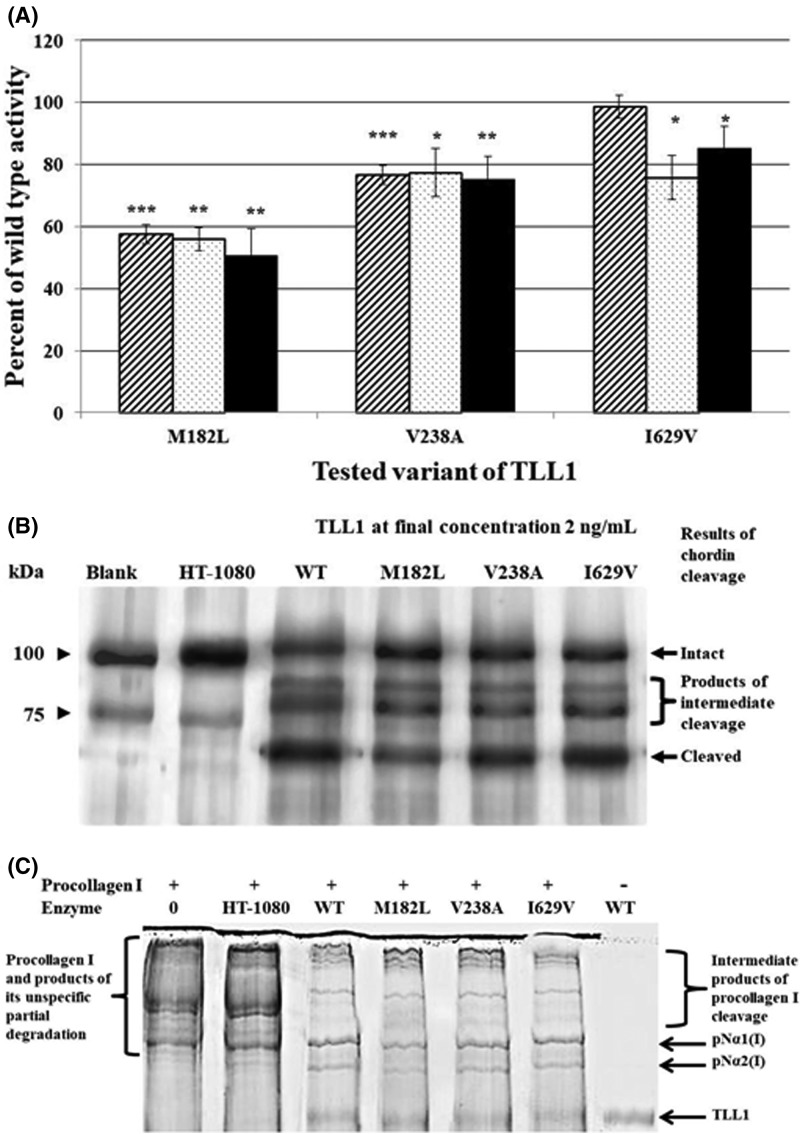
TLL1 enzymatic activity tests against synthetic labeled peptide, chordin, and procollagen I. The cleavage of chordin and procollagen were detected by electrophoresis of the reaction products following their incubation with recombinant wild-type or mutated variants of TLL1 (**A**) Lowered cleavage rates for the mutated variants of TLL1. The histograms represent percent of wild-type TLL1 activity on fluorescently labeled peptide MCA-YVADP-DNP-K – slashed filed boxes, chordin – doted boxes and procollagen I – black boxes. Statistically significant difference between wild-type and mutated variants are indicated by asterisks: *P<0.05, **P<0.01, ***P<0.001. (**B**) Results of cleavage of chordin: lanes from left contained reaction products of chordin incubated in the enzymes absence (Blank) or in the presence of: proteins obtained from culture medium of mock transfected HT-1080 cells (HT-1080), purified recombinant enzymes of wild-type (WT) or its mutated variants (M182L, V238A, I629V). (**C**) Pattern of electrophoretic separation of the cleavage products of procollagen I in the presence of wild type TLL1 and its mutated variants. Lanes contained: molecular weight marker (M) and procollagen I incubated without enzymes (0), and with proteins obtained from culture medium of mock transfected HT-1080 cells (HT-1080), purified recombinant enzymes of wild-type (WT) or its mutated variants (M182L, V238A, I629V). Abbreviations: pNα1(I) – α 1 chain with amino-terminal propeptide of collagen type I; pNα2(I) – α 2 chain with amino-terminal propeptide of collagen type I.

The fluorescence intensity depending on enzyme concentration was linear for all tested TLL1 variants. The plateau of the peptide cleavage kinetics for wild-type TLL1 and the I629V mutant were the same, and they were reached for each enzyme at concentrations of approximately 12 ng/μl. The cleavage kinetics by mutated variants M182L and V238A were lower and constituted ∼57 and ∼77% of the cleavage by the wild-type TLL1, respectively (Figure 4A).

### Enzymatic activity assay of native and mutated TLL1 enzymes with chordin substrate

Enzymatic activity test of the TLL1 and its variants on chordin revealed specific cleavage of this substrate by all tested variants, similar to the wild-type TLL1 (Figure 4B). Products in the control blind sample and the sample treated with culture medium proteins obtained from mock transfected HT-1080 cells revealed the banding patterns as expected for non-cleaved chordin. For chordin incubated with the wild-type TLL1 as well as with its mutated variants, the banding patterns following their electrophoresis consisted of four bands. Two bands corresponded to the peptides detected in the control samples, and two were the intermediate product of chordin cleavage without the C-terminal portion and fully processed chordin lacking both C- and N- termini (Figure 4B).

Differences in the cleavage rates for wild-type TLL1 and its mutated variants were detected as lower rates resulting in higher density of bands corresponding to non-cleaved peptides marked as ‘Intact’ in Figure 4B. Pixel densities of the band corresponding to the ‘Intact’ reaction product retained in the presence of the mutated variants M182L, V238A, and I629V were higher than in the presence of wild-type TLL1, therefore, the activities of the mutated variants were lower by 50% for M182L and ∼25% for V238A and I629V than for the wild-type TLL1 (Figure 4A).

### Enzymatic activity assay of native and mutated TLL1s with procollagen type I as the substrate

Following electrophoresis of 500 ng of procollagen type I incubated without enzyme, a visible, intense band and some bands corresponding to its partial degradation were detected in the expected range (Figure 4C). In samples subjected to incubation with TLL1 and its mutated variants the major band corresponding to procollagen I fade and bands identified as pN-collagen α chains were detected. However, also products corresponding to partial enzymatic processing of procollagen I, which is a characteristic of collagen maturation were detected (Figure 4C). In the lane containing products of incubation in the presence of the proteins purified from the culture medium of the mock transfected HT-1080 cells, no bands characteristic for the procollagen I cleavage by TLL1 could be detected (Figure 4C). In lanes containing products of procollagen I incubation in the presence of TLL1, increased density of bands corresponding to pN-collagen I α 1 and 2 chains, as the results of procollagen I processing were detected in expected ratio close to 2:1 of pNα1 to pNα2, (Figure 4C). This correlation was found both for wild-type and mutated variants of TLL1. However, for the mutants, the rates of procollagen I cleavage were slower. As stated in the ‘Materials and methods’ section, the band corresponding to unreduced triple-helical procollagen type I was of poor resolution, therefore densities of bands corresponding to the pNα1(I) and pNα2(I) were measured (Figure 4C). The TLL1 mutated variants samples M182L and V238A, as well as I629V, contained less of the products corresponding to measured bends, thus indicated lowered cleavage rates than the wild-type by respectively: 50, 25, and 15% (Figure 4A).

### Detection of higher order peptide structures in TLL1 and its mutated variants by CD spectrometry

**Figure 5:**
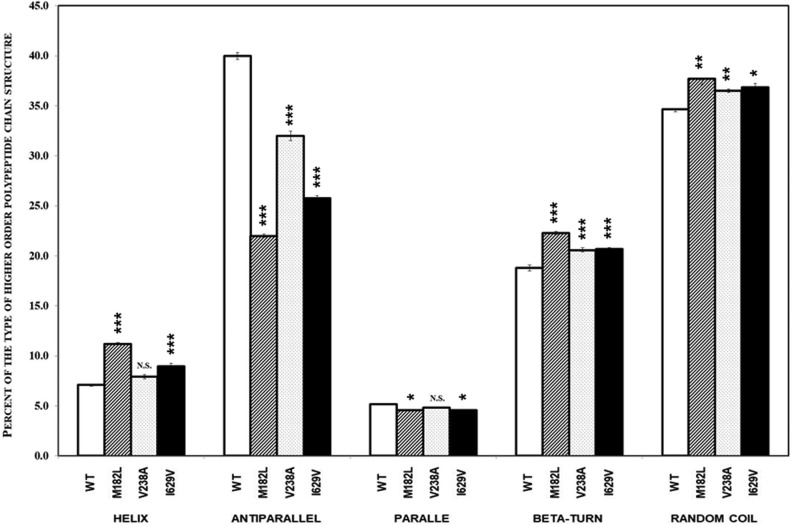
Percent of the polypeptide structure content in TLL1 and its mutants determined by CD spectrometry measurements Empty boxes – TLL1, slashed boxes – M182L, hatched horizontally boxes – V238A, Filled boxes – I629V. The statistically significant differences (*P*<0.05 - “*”; *P*<0.01 - “**”; *P*<0.001 - “***”) in structures content between TLL1 and its mutated variants are indicated above the boxes.

The overall structure of tolloids is assumed to be similar to astacin in their catalytic domains and to structures formed by sequences found in Complement and sea urchin and BMP1 (CUB) and by sequences present in Epidermal Growth Factor (EGF) domains forming their regulatory parts [30,33,34]. Therefore, the secondary structures comprising TLL1 are helix – 7.1%, antiparallel – 39.99%, parallel – 5.19%, β-turn – 18.80%, and random coil – 34.64%. Analyses of the CD spectra revealed statistically significant differences in the content of the secondary structures between wild-type and the TLL1 mutants. The major difference was detected in the content of antiparallel structures between the wild-type TLL1 and all of the mutated variants (Figure 5).

A difference for antiparallel structure content was detected between the wild-type TLL1 and the mutant M182L (Figure 5). Smaller differences in the content of these types of structures were found between the wild-type TLL1 and mutant M238V. The third mutant had changed leucine at position 629 to valine within the sequence forming the CUB3 domain. This residue is a part of β-turn 3, one of ten such structures resembling a jelly-roll-type structure [30].

### The binding affinity of TLL1 and its mutated variants to chordin

Biosensor assays have demonstrated that all of the mutations introduced into TLL1 decreased the affinity of mutants for binding to chordin (CH) (Table 1). This may indicate that the mutations both in the catalytic domain as well as in the regulatory CUB3 domain affected the interactions between the mutated metalloendopeptidases and chordin, resulting in lower cleavage rates.

**Table 1:** Results of chordin binding with TLL1 and its mutated variants

Binding interaction	k_on_ [M^−1^.s^−1^]	*k*_off_ [s^−1^]	*K*_d_ [M]	*K*_a_ [M^−1^]
WT-CH	2.6 × 10^3^	3.05 × 10^−4^	1.17 × 10^−7^	8.6 × 10^6^
M182L-CH	4.3 × 10	1.8 × 10^−3^	4.2 × 10^−5^	2.4 × 10^4^
V238A-CH	3.3 × 10	6.0 × 10^−4^	1.8 × 10^−5^	5.5 × 10^4^
I629V-CH	3.7 × 10	9.1 × 10^−4^	2.5 × 10^−5^	4.1 × 10^4^

## Discussion

Thus far, experimentally confirmed substrates for TLL1 in addition to chordin are procollagen types I, II, and VII [35,36], lysyl pro-oxydase [37,38], osteoglycine [39], decorin [40], probiglycan [41], and perlecan [42]. Additionally, some of its reported substrates were molecules involved in intercellular signaling, such as prolactin [43], myostatin [44], and neuralin [45]. The murine Tll^−/−^ heart abnormalities reported by others [19] mimic those found in ASD6 in human, which is an inborn defect. Therefore, we analyzed the effect of the three mutations on chordin, the natural TLL1 substrate. Additionally, the original *in vitro* work by Takahara et al. [18] reported variable activity of mammalian BMP-1/Tolloid-related metalloproteinases on procollagen type I as possible substrate of TLL1. In our work, we limited the test of cleavage rates by the three mutated variants to these two substrates as both are important for heart development. Procollagen type I is the most abundant ECM structural component and chordin is an important link in regulation of BMP-related signaling and both are putative TLL1 substrates.

It is commonly accepted that ECM structure is essential for the proper course of the EMT, proliferation, and differentiation of cardiomyocytes [46–48]. In humans, mutations in the collagen genes, mainly type I, reduce either the formation or the stability of collagen fibers [49,50]. However, in patients with collagen I related disorders, cardiac abnormalities are rarely seen and mainly consist of mitral and aortic valve insufficiency, as a consequence of the weakened ECM structure [51,52]. Also, in the transgenic Tll^−/−^ mice no collagen fibril abnormalities were detected indicating that procollagen type I is not TLL1 natural substrate of this species [19].

Several other substrates for TLL1 were proposed based on *in vitro* enzymatic assays. One such TLL1 substrate is probiglycan, which participates not only in the formation of the ECM but also in the intracellular signaling path in conjunction with TGF-β, nitric oxide (NO) and regulation of intracellular Ca^2+^ level [53,54]. In biglycan knockout mice, aortic rupture was observed as a result of disorders of the ECM, resulting in a reduction in the flexibility and strength of the connective tissue [55]. The presence of biglycan is essential to maintain the phenotype of cardiofibroblasts [56]. Biglycan also supports the regeneration of the heart after myocardial infarction [57].

Another putative substrate of TLL1, decorin, regulates fibrylogenesis and is also an inhibitor of TGF-β signaling [58]. It has a protective effect against the development of fibrosis in the heart [59]. Studies in a chicken embryo have shown that decorin deficiency resulted in deformation of embryonic blood vessels and thinning of the ECM in the developing heart. In addition, decorin is important for the migration of neural crest cells [60].

Another potential TLL1 substrate, perlecan, a component of basement membranes, is essential for the structural stability of the heart. Studies in transgenic mouse embryos with perlecan knockout revealed that the walls of developing hearts exhibited mechanical instability on day 10.5 of embryo development. This was the result of a defective structure of the basement membrane, which was lower in the basic components such as collagen IV and laminin.

In connection with *BMP1* gene expression, the structure of the ECM in *tll1*^−/−^ mice did not differ from that observed in the wild-type, suggesting that the phenotype of *tll1*^−/−^ mice is not an effect of irregularities in this area. However, changes in the hearts of *tll1*^−/−^ mice were related mainly to these structures, within which the expression of *tll1* and *bmp1* does not overlap, indicating the possibility of formation of regions with different structures of ECM [20]. However, in view of the above facts, the phenotype of *tll1*^−/−^ mice seems unlikely to be directly caused by changes in the structure of the ECM resulting from the lack of cleavage of the previously listed substrates. Therefore, it should be considered whether the lack of TLL1 affects the signaling processes that contribute to the differentiation and proliferation of heart cells.

The TLL1 substrate osteoglycine, which is primarily responsible for mineralization of bone tissue, also plays a role in heart development. It has been shown that osteoglycine regulates the muscle mass of the left ventricle of the heart in the rat, mouse, and human, and its overexpression is associated with the occurrence of cardiac hypertrophy [61]. This is because osteoglycine stimulates proliferation of cardiomyocytes. Therefore, in the case of *tll1*^−/−^ mice, a deficit of osteoglycine mature form could arise, which in turn could lead to slower proliferation of cells forming the septa of the heart.

Two other substrates, perlecan and prolactin, are involved in the regulation of angiogenesis. As a result of cleavage of these substrates by tolloids, including TLL1, products are released that have anti-angiogenic properties. Therefore, it seems that the role of the tolloids is anti-angiogenic. However, blocking their activities inhibited the growth of blood vessels in chick embryos. *In vitro* studies have shown that this is due to the reduction in the rate of endothelial cell proliferation [62].

The expression of chordin was present in the heart of the mouse embryos, although it was not the highest expressing tissue. The highest expression level of chordin in the heart was observed at day 8.5 of embryonic development, after which it falls rapidly to the limits of detection, between day 9.5 and 13.5 of embryonic development. The expression of *tll1* varied from barely detectable level at day 8.5 to its rapid increase between day 9.5 and 11.5, with the highest level at day 13.5 of embryonic development. Thus, the expression of *tll1* and chordin in the heart during embryonic development in the mouse had a reverse time pattern. During the period when malformations of the heart emerged, the characteristic phenotype of *tll1*^−/−^ mice for chordin expression was the lowest, whereas the *tll1* expression was the highest, indicating a significant BMP2/4 mobility at that time [19]. In addition, in mice with knockout of the gene encoding chordin, a phenotype was observed that is characteristic of Di George syndrome in humans. These include such irregularities as the lack of the thymus, distortion of the larynx, and a cleft palate, including an incomplete septal [63].

Lower cleavage rates of chordin by the all three mutants in humans support the concept that chordin, through its participation in signaling involving BMP2/4, seems to be the most matched substrate for TLL1. Therefore, it supports previous suggestions that TLL1 could be responsible for the phenotype of *tll1*^−/−^ mice as well as ASD6 in humans. If the cleavage of other discussed putative TLL1 substrates is also affected by the mutation, the final ASD6 pattern of the heart could result from lower cleavage rates as we have observed for chordin. Particularly the mutations M182L and V238A localized within the catalytic domain recognizing the correct cleavage site might affect the cleavage of the wide range of TLL1 putative substrates discussed here. Additionally partial intracellular retention of M182L mutant that could be explained by previous observations from experiments in which CUB1 and other CUB domains were interchanged, resulted in retention of the proteins by cells. Therefore, CUB1 and its location immediately adjacent to the metalloproteinase domain are essential for TLL1 secretion [64]. Putative retention of the M182L mutant together with shown in our test lowered cleavage rate indicate that this mutation could contribute to development of ASD6.

The analyses of secondary and tertiary structures by CD spectrometry indicated that these mutations affected neighboring conformations. The amino acids replaced in mutants M182L and V238A are respectively in the antiparallel helices 2 and 3 that form the active center in the TLL1 catalytic domain. Since the peptidolysis of synthetic substrate by the two mutants was weaker, the two helices seem to be crucial for docking the peptide fragment containing the peptide bond recognized by the peptidase. The third mutant with the change in CUB3 domain cleaved the synthetic peptide as the wild-type did. The amino acid change in the third mutant is placed in a signature GX(I/L)T(S/T)PGWPKEYPNK, which is located in CUB3. The entire signature is highly conserved amongst the CUB2, 3, and 4 domains in TLD and other TLLs with isoleucine preserved in all of the CUBs except in CUB4 of TLL2. This indicates the functional importance of this peptide in the overall structure of this protein. The significance of the change in CUB3, although it did not affect its catalytic action on a synthetic substrate, did significantly decrease cleavage of the two natural substrates—the specific one, chordin, and the less specific one, procollagen type I. The statistically significant difference in the content of secondary antiparallel structures in this mutant in comparison with the wild-type supports the putative role of this domain in recognition by TLL1 of the specific substrates.

In conclusion, (i) the mutation V238A caused partial retention of the protein in the cell cytoplasm and expressed a lower cleavage rate of chordin, indicating a potential causative role in the development of the ASD6 condition. (ii) The mutation M182L affects spatial enzyme structure, thus affecting its enzymatic activity, and plays a role in development of ASD6. (iii) Although purified recombinant enzymes all show activity on tested substrates, (a) the mutated variants (M182L, V238A and I629V) had lowered enzymatic activity rates on chordin and procollagen C-proteinase type activity on procollagen I. (b) Mutations M182L and V238A possibly restricted TLL1 activity due to changes in the catalytic domain. (c) Lower activity of mutant I629V was not related to changes in the TLL-1 catalytic center but rather was a result of weaker affinity to substrates or stronger homodimerization of the enzyme. (d) Spatial structure of the mutants was disturbed, and the specificity of site recognition, surrounding the hydrolyzed peptide bound in the substrates, was also jeopardized.

## Supporting information

**Table S1.** The list of primers used and their characteristics.

## Abbreviations

AB: assay buffer
ASD6: atrial-septal defect 6
BMP1: bone morphogenetic protein 1
CMV: Cytomegalovirus
CUB: Complement and sea urchin and BMP1
DMEM: Dulbecco’s Modified Eagle’s medium
ECM: extracellular matrix
EMT: epithelial–mesenchymal transition
HBS-TE: HEPES-buffered saline containing 0.005% of Tween 20
IRES: Internal Ribosome Entry Site sequence
LRG1: leucine rich alpha-2-glycoprotein
OMIM: Online Mendelian Inheritance In Man
SMAD1: Mothers against decapentaplegic homolog 1
TAE: Trizma acetate EDTA buffer
TGF-β: Transforming growth factor-beta
TLD: mammalian Tolloid
TLL1: tolloid-like 1
TLL2: tolloid-like 2
TSF1-8: Name for froward primers numbered from 1 to 8
TSR1-8: Name for reverse primers numbered from 1 to 8
VSD: ventricular septal defect
