# Supplementary material for: Functional and structural studies of tolloid-like 1 mutants associated with atrial-septal defect 6
Source: Biosci Rep. 2019 Jan 8;39(1):BSR20180270. doi: 10.1042/BSR20180270 (PMC6328869; doi:10.1042/BSR20180270)
Supplement: Supplementary file 1 [file bsr20180270_Supp1.pdf]

Supplemental material

Functional and structural studies of tolloid-like 1 (TLL1) mutants associated with atrial-septal defect 6 (ASD6).

Table S1. The list of primers used and their characteristics.

**List of oligonucleotides used for cDNA amplification**		
**Name**	**Sequence**	**Use**
**A1F**	AGGCAGTGCTTGCCCTCTCTA	First PCR for amplification A TLL-1 fragment
**TSR3**	GCATTCTTTCCTGCTTGGGTACTT	
**TSF3**	GGAGATATCGCACAGGCAAGAA	First PCR for amplification B fragment TLL-1
**TA1BR**	CTGGCCTGCAAGTGGAAATAATGA	
**FASTCOL**	GACGCACGGCGCCGCTACATCAAGAACGGAAAGAA	Second amplification of B fragment TLL-1, introducing *Nar*I site at 5'-end
**RW1SECO**	CAGATTTGAATTCAATTCTCA	
**FW1SECO**	TGAGAATTGAATTCAAATCT	Second amplification of A fragment TLL-1, introducing 7 histidine tag on 3'-end
**RW1HISN**	AAGCGGCCGCTTAATGATGATGATGATGATGATGTTTTTTGGTATGTGTGGTATCTGG ATATCTT	
**COLZ**	TCGGAGCAGACGGGAGTTTC	Amplification of C fragment, introducing *Nar*I at 5'
**RCOLAST**	TCTTTCCGTTCTTGATGCGGCGCCGTGCGTCAGGAGGG	
**FCW1PINA**	TTTTACCGGTGCCGCCATGTTCAGCTTTGTGGACCTCCG	Amplification of C-A fragment (with RW1SECO), introducing *Pin*AI site at 5'-end
**IRSEGF**	ATGTCATGCCCGCTTTTTGAG	Colony PCR and plasmid sequencing
**NEO3R**	ATGGGCGGTAGGCGTGATA	
**M1F**	TGAGGCACTGGGAAAAGCACACAT	Introducing mutation M182L
**M1R**	GGGCCTGCTTGAACATGGCTCT	
**M2F**	TTGGGATTGCTGTTCATGA	Introducing mutation V238A

**M2R**	TGAACAGCAATCCCAAATT	
**M3F**	CGGCACCGTAACCACCCCT	Introducing mutation I629V
**M3R**	CCAGGGGTGGTTACGGTGCCGT	
**TA1F**	AGGCAGTGCTTGCCCTCTCTA	Sequencing of the constructs
**TA1R**	ATGCTGATGAGGGTTTTGCTGGAAA	
**TSF1**	ACATACCACAGGTGGACTTGGA	
**TSF2**	CGAGGAAATGGACCTCAGGCAA	
**TSF3**	GGAGATATCGCACAGGCAAGAA	
**TSF4**	GCAGCAGTAATTGGGTAGGAAA	
**TSF5**	AGAGGAAGATGAGTGTGCCAAA	
**TSF6**	GGCAAATTCTGTGGCGCTGAA	
**TSF7**	ACTCCTGGCCACCGAATCAAA	
**TSF8**	AACGGGGCTCTCGACTTGAA	
**TSR1**	TTCCAAAGGGGTTCTGCGTAA	
**TSR2**	AGCAGCATCCACAAGGCCTATA	
**TSR3**	GCAGGACGTATGCCATTATCATCA	
**TSR4**	ACAGAATCTACCAAGGAGAGGTGATT	
**TSR5**	TCGCCCTATCAAAGGGCTATTTT	
**TSR6**	CCACTCCAGATCTCCACATAATCTA	
**TSR7**	GCATTCTTTCCTGCTTGGGTACTT	
**TSR8**	CAACCTGTCCTGGGTAGTTGTTA	
